# Developing and validating a clinlabomics-based machine-learning model for early detection of occult diabetic kidney disease: implications for primary care screening

**DOI:** 10.3389/fendo.2026.1835866

**Published:** 2026-04-27

**Authors:** Mengyu Zhang, Sudi Zhu, Di Hu, Wenqiang Fu, Henggui Hu, Yuanyuan Xu, Kaixuan Zhang, Heng Tang, Xiaolei Du

**Affiliations:** Department of Clinical Laboratory, Wanbei Coal Electric Group General Hospital, Suzhou, China

**Keywords:** clinlabomics, machine learning, occult diabetic kidney disease, primary care, risk stratification, uric acid

## Abstract

**Background:**

Occult diabetic kidney disease (DKD) is a subtle yet high-risk microvascular complication of type 2 diabetes mellitus (T2DM). Early-stage DKD often goes undetected because traditional screening markers remain within the normal range. This study aimed to develop and validate an explainable machine learning (ML) model using routine clinical and laboratory data for the early detection of occult DKD. Its potential value for primary care screening was also evaluated.

**Methods:**

This multicenter retrospective study included 1,916 hospitalized patients with T2DM. The derivation cohort consisted of 1,066 patients from Wanbei Coal-Electricity Group General Hospital and was used to train the model. An independent cohort of 850 patients from the First Affiliated Hospital of Anhui Medical University served for external validation. Thirty-two routine clinical variables were initially considered. Eight ML algorithms were compared to identify the optimal model. SHapley Additive exPlanations (SHAP) was employed to rank feature importance, reduce variables, and interpret the model. Finally, a quartile-based risk stratification system and a web-based tool were developed.

**Results:**

Among the eight algorithms, logistic regression (LR) showed the best performance. Using SHAP rankings, a simplified LR model was built with eight features: HGB, HbA1c, HTN, UA, sex, MicroVCs, CVD, and A/G. The model performed well in both the training cohort (AUC = 0.824) and the external validation cohort (AUC = 0.786). SHAP analysis identified HbA1c, uric acid (UA), and hemoglobin (HGB) as the top contributors. The risk stratification system demonstrated clear separation, with the incidence of occult DKD rising from 1.5% in the lowest-risk quartile (Q1) to 55.8% in the highest-risk quartile (Q4). Additionally, decision curve analysis demonstrated that the model provides substantial clinical net benefit, and the final model was implemented as an interactive web-based calculator for real-time risk assessment.

**Conclusion:**

An explainable ML model was successfully developed to accurately predict occult DKD using routine clinical data. The model combines good performance with clear interpretation. It may serve as a practical tool for large-scale screening and early intervention in primary care.

## Introduction

Diabetic kidney disease (DKD) is one of the most severe microvascular complications of type 2 diabetes mellitus (T2DM). It remains the leading cause of end-stage renal disease (ESRD) worldwide (1). Clinical diagnosis and monitoring of DKD have traditionally relied on two markers: an elevated urinary albumin-to-creatinine ratio (UACR) and a reduced estimated glomerular filtration rate (eGFR) (2). However, in routine primary care, a misleading clinical scenario frequently arises so-called “occult” diabetic kidney disease (occult DKD). When a patient’s eGFR remains within the normal range (a 60 mL/min/1.73 m²), physicians often assume that renal function is preserved and consequently forgo urinary albumin testing (UACR) (3, 4). Because early-stage occult DKD is often asymptomatic, it is frequently missed in routine practice. This delay in detection can lead to late intervention and a marked increase in both cardiovascular and all-cause mortality (5).

Currently, the clinical diagnosis of DKD mainly relies on UACR and eGFR (2). However, these markers have clear limitations for early detection and prevention (6). Changes in UACR and eGFR usually occur only after substantial and often irreversible renal damage, such as podocyte injury, mesangial expansion, and tubulointerstitial fibrosis (5). In other words, these indicators reflect relatively late stages of the disease. By the time they become abnormal, the optimal window for early intervention may have already passed. This diagnostic gap directly contributes to the occurrence of “occult” DKD. Because UACR testing is often underutilized when serum creatinine and eGFR appear normal, early microalbuminuria (UACR ≥ 30 mg/g) may go undetected in routine clinical practice, leading standard screening approaches to miss high-risk patients (3). To address these gaps, researchers have investigated new early biomarkers related to DKD pathogenesis. These include lipid-related markers, such as the Atherogenic Index of Plasma (AIP) (7, 8); inflammatory indicators, such as the neutrophil-to-lymphocyte ratio (NLR) (9, 10); and measures of glycemic control, including HbA1c and glycated albumin. However, due to the complex and heterogeneous nature of DKD, no single biomarker has demonstrated sufficient sensitivity and specificity (11). Therefore, there is a clear need for a comprehensive risk model. A “clinlabomics” approach, which integrates multiple routine clinical and laboratory variables, may provide a practical and effective solution (12).

In recent years, machine learning (ML) has transformed clinical decision-making. It is particularly suited for handling high-dimensional data and can uncover patterns often missed by traditional statistical methods (13–15). Although several ML models have been developed for DKD prediction, important gaps remain (16). First, most studies focus on overt DKD, while the more insidious occult DKD is largely overlooked. Second, many models operate as “black boxes” and lack the transparency required for clinical use (17). Third, few studies provide practical tools, such as interactive calculators, or include large-scale external validation, limiting their real-world applicability (16).

To address these gaps, we conducted a multicenter retrospective study including 1,916 hospitalized patients with T2DM. The study aimed to develop and validate a clinlabomics-based ML model for the early detection of occult DKD, clarify feature importance, and interpret the optimal model using the SHAP method (17). We also assessed its prognostic value by establishing a four-tier risk stratification system and an interactive web-based tool. Ultimately, this framework offers a practical and accessible approach to support large-scale screening and decision-making in primary care.

## Methods

### Study population

Clinical and laboratory data of hospitalized patients diagnosed with T2DM were retrospectively collected from two independent tertiary medical centers: Wanbei Coal-Electricity Group General Hospital (training cohort) and the First Affiliated Hospital of Anhui Medical University (external validation cohort) between January 2023 and December 2025. After applying strict inclusion and exclusion criteria, a total of 1,916 eligible patients were enrolled. The study was conducted in accordance with the principles of the Declaration of Helsinki and was formally approved by the Ethics Committee of Wanbei Coal-Electricity Group General Hospital (WBZY-LLWYH-2026-001). Given the retrospective design and the use of fully anonymized patient data, the requirement for written informed consent was waived by the institutional review board.

### Inclusion and exclusion criteria

The inclusion criteria were defined as follows: (1) adult patients aged 18 years or older, and (2) a confirmed diagnosis of T2DM according to the World Health Organization (WHO) or American Diabetes Association (ADA) criteria.

To ensure a well-defined study population and minimize potential confounding, patients were excluded if they met any of the following criteria: (1) acute kidney injury (AKI) or primary glomerular diseases (e.g., IgA nephropathy or membranous nephropathy); (2) overt diabetic kidney disease; (3) severe hepatic dysfunction, active malignancy, or acute infection; (4) pregnancy or lactation; or (5) substantial missing clinical or laboratory data.

### Definition of occult DKD

As the core focus and distinguishing feature of this study, occult DKD was strictly defined to capture the ultra-early, asymptomatic stage of diabetic renal injury (18). Specifically, patients were classified as having occult DKD if they had a confirmed diagnosis of T2DM, preserved renal function, and evidence of early microalbuminuria. Preserved renal function was defined as an eGFR ≥ 60 mL/min/1.73 m², calculated using the Chronic Kidney Disease Epidemiology Collaboration (CKD-EPI) equation, while early microalbuminuria was defined as a UACR ≥ 30 mg/g. In contrast, the non-occult DKD control group comprised patients with T2DM who had both preserved renal function (eGFR ≥ 60 mL/min/1.73 m²) and normoalbuminuria (UACR < 30 mg/g). This stringent definition ensures that the predictive model specifically targets this clinically elusive phenotype, enabling the identification of high-risk patients at a stage when routine functional markers (eGFR) remain within the normal range and UACR testing is often overlooked. This approach may facilitate truly early clinical intervention.

### Data collection and processing

All demographic characteristics, clinical histories, and laboratory data were retrospectively extracted from the electronic medical record (EMR) systems of the participating centers. To accurately capture patients’ baseline physiological and metabolic status, laboratory parameters were primarily obtained from the first fasting blood samples collected within 24 hours of hospital admission. A detailed description of all extracted variables, including definitions and measurement units, is provided in **Supplementary Table 1**.

Given the pivotal roles of systemic low-grade inflammation and lipid metabolism disorders in DKD pathogenesis, several composite clinlabomics indices were calculated using standard formulas. These included: the triglyceride-glucose (TyG) index, calculated as ln(TG × GLU/2) (19); the AIP, calculated as log_10_(TG/HDL-C); the NLR, calculated as neutrophils/lymphocytes; the platelet-to-lymphocyte ratio (PLR), calculated as platelets/lymphocytes; and the systemic immune-inflammation index (SII), calculated as (platelets × neutrophils)/lymphocytes (20).

Rigorous data cleaning and preprocessing were performed to ensure dataset quality. First, to minimize potential bias and distortion from missing data, any feature with a missing value rate exceeding 25% was excluded from the analyses. Among the remaining variables, the overall proportion of missing data was low, accounting for less than 5% of the dataset. Residual missing values were imputed using the median to preserve cohort structure and maintain statistical power. Second, because multicollinearity among variables can destabilize machine learning algorithms and reduce predictive accuracy, Spearman’s rank correlation analyses were conducted to assess pairwise correlations among continuous features (**Supplementary Figure 1**). When two features were highly correlated (correlation coefficient > 0.6), the feature deemed less clinically relevant to renal outcomes was removed.

Following these rigorous screening and preprocessing steps, a total of 32 clinlabomics features were retained for the initial development of the prediction models. These features were grouped into three categories: (1) Demographics and medical history: age, sex, hypertension (HTN), hyperlipidemia, cardiovascular disease (CVD), and microvascular complications (MicroVCs). (2) Routine biochemical and hematological parameters: albumin-to-globulin ratio (A/G), albumin (ALB), alkaline phosphatase (ALP), blood urea nitrogen (BUN), fasting glucose (GLU), high-density lipoprotein (HDL), low-density lipoprotein cholesterol (LDL-C), prealbumin (PAB), total bilirubin (TBIL), triglycerides (TG), total protein (TP), uric acid (UA), basophil count (BASO#), eosinophil count (EO#), HGB, lymphocyte count (LYMPH#), monocyte count (MONO#), neutrophil count (NEUT#), platelet count (PLT), glycated hemoglobin (HbA1c), and gamma-glutamyl transferase (GGT). (3) Calculated composite indices: NLR, platelet-to-lymphocyte ratio (PLR), systemic immune-inflammation index (SII), triglyceride-glucose index (TyG), and AIP.

### Model development and comparison

Data from Wanbei Coal-Electricity Group General Hospital (n = 1,066) were used exclusively for model derivation. An independent dataset from the First Affiliated Hospital of Anhui Medical University (n = 850) served as an external validation cohort to assess model generalizability. The 32 clinlabomics features described above were used to develop the prediction models. Missing data were handled using median imputation (21), and detailed variable descriptions are provided in **Supplementary Table 1**. Eight machine learning models were applied to predict the risk of occult DKD in patients with T2DM: decision tree (DT), K-nearest neighbor (KNN), light gradient boosting machine (LightGBM), LR, naive Bayes (NB), random forest (RF), support vector machine (SVM), and eXtreme gradient boosting (XGBoost). Hyperparameters for each model were optimized in the training cohort through a combination of grid search, manual fine-tuning, and five-fold cross-validation to prevent overfitting. Model performance was evaluated using standard metrics, including the area under the receiver operating characteristic curve (AUC), sensitivity, specificity, positive predictive value (PPV), negative predictive value (NPV), accuracy, and F1 score. Detailed specifications regarding the hyperparameter space, random seeds, and software environments for all evaluated algorithms are provided in **Supplementary Table 5**.

### Feature selection and model explanation

The initial set of 32 clinlabomics variables underwent a rigorous dimensionality reduction process guided by SHAP importance rankings. Features were removed sequentially from the baseline model. DeLong’s non-parametric test (22) was used to compare the AUC between the baseline model and each progressively simplified version. Feature elimination was stopped once a significant decrease in AUC was observed (*P* < 0.05), resulting in an optimal subset of eight features.

A forest plot was used to display the independent prognostic value of the finalized predictors. The SHAP framework provided comprehensive interpretability for the model. Global SHAP analyses quantified the overall contribution and directional effect of each retained feature on the risk of occult DKD, while local SHAP evaluations illustrated personalized prediction trajectories for individual patients based on their clinical profiles. Finally, a clinical risk stratification system was developed using predicted probabilities from the optimal model to support triage and decision-making in primary care.

### Web-based model deployment

The optimal machine learning model, incorporating the finalized eight clinlabomics features, was deployed as an interactive web-based application using the open-source Streamlit framework. This digital interface allows clinicians to input routine patient parameters and immediately obtain a personalized risk probability for occult DKD. By translating complex algorithmic calculations into an accessible format, the online calculator facilitates real-time decision-making and early triage in primary care settings.

### Statistical analysis

Continuous variables are presented as medians with interquartile ranges (IQRs), and categorical variables are summarized as frequencies and percentages. Group comparisons were performed using the Mann-Whitney U test for non-normally distributed continuous variables, and the Chi-square or Fisher’s exact test for categorical variables. All machine learning model development and graphical visualizations including receiver operating characteristic (ROC) curves, decision curve analysis (DCA), precision-recall (PR) curves, heatmaps, and forest plots were conducted using Python (version 3.11) within the PyCharm integrated development environment (version 2023.3.5). Statistical significance was defined as a two-sided *P* < 0.05.

## Results

### Patient characteristics

**Figure 1:**
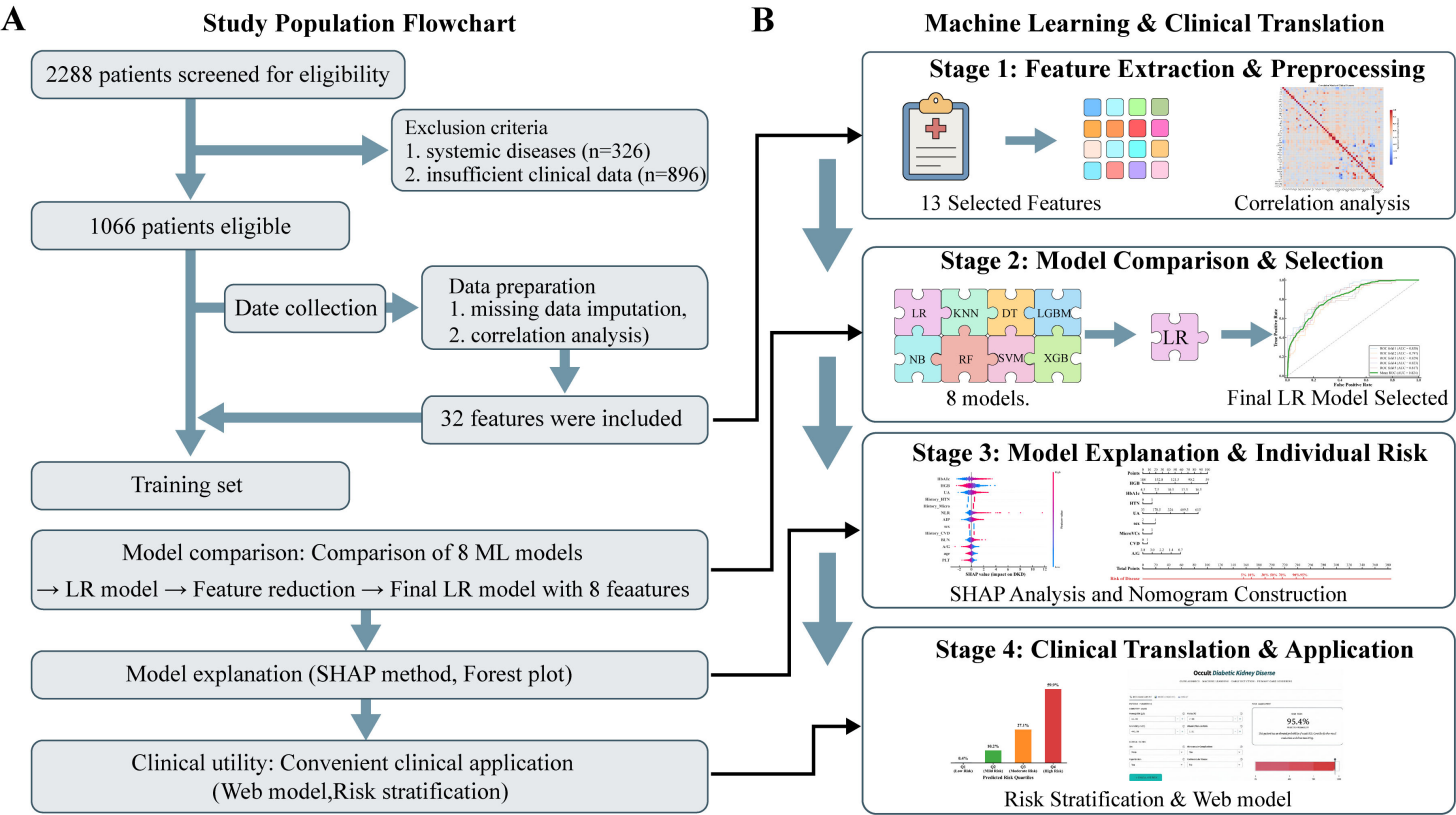
Workflow for predicting occult DKD via clinlabomics combined with machine learning. **(A)** Flowchart detailing patient screening, exclusion criteria, and the final derivation of the training cohort. **(B)** The four-stage machine learning pipeline encompassing feature preprocessing, comparison of eight predictive models, SHAP-based model interpretability analysis, and the development of clinical translational tools (web-based calculator and risk stratification). DT, decision tree; KNN, k-nearest neighbor; LGBM, light gradient boosting machine; LR, logistic regression; NB, naive Bayes; RF, random forest; SHAP, SHapley Additive exPlanations; SVM, support vector machine; XGBoost, extreme gradient boosting.

A total of 1,916 eligible patients diagnosed with T2DM were included in this retrospective study. The population was divided into a training cohort (n = 1,066) from Wanbei Coal-Electricity Group General Hospital and an independent external validation cohort (n = 850) from the First Affiliated Hospital of Anhui Medical University. The dual-panel study design, including the patient screening flowchart and the machine learning workflow, is shown in **Figure 1**. Baseline demographic, clinical, and laboratory characteristics of the training cohort are summarized in **Table 1**, while corresponding data for the external validation cohort are provided in **Supplementary Table 2**. Specifically, the prevalence of occult DKD was 24.4% (260/1066) in the training cohort and 43.1% (366/850) in the external validation cohort.

**Table 1:** Baseline demographic, clinical, and laboratory characteristics of the training cohort.

Characteristic	Label		*p*-value
	Non-occult DKD N = 806	occult DKD N = 260	
age, year	58 (52, 65)	59 (53, 70)	0.022
Male, n (%)	438 (54.3%)	159 (61.2%)	0.054
A/G	1.67 (1.52, 1.88)	1.59 (1.42, 1.78)	<0.001
ALB, g/L	42.3 (40.1, 44.6)	40.7 (38.0, 43.8)	<0.001
ALP, U/L	77 (66, 93)	80 (68, 99)	0.017
BUN, mmol/L	5.77 (4.80, 6.80)	6.49 (5.07, 7.76)	<0.001
GLU, mmol/L	8.5 (6.4, 10.0)	9.1 (7.6, 11.8)	<0.001
HDL, mmol/L	1.15 (0.98, 1.41)	1.08 (0.89, 1.29)	<0.001
LDL-C, mmol/L	2.49 (1.92, 3.13)	2.45 (1.87, 3.04)	0.460
PAB, mg/L	231 (213, 253)	231 (197, 245)	0.006
TBIL, μmol/L	13.7 (10.7, 17.3)	13.0 (10.0, 16.7)	0.033
TG, mmol/L	1.48 (1.06, 2.14)	1.80 (1.15, 2.82)	<0.001
TP, g/L	67.3 (64.1, 71.0)	67.3 (62.5, 71.0)	0.154
UA, μmol/L	266 (219, 316)	307 (249, 364)	<0.001
BASO#, 10^9^/L	0.030 (0.020, 0.040)	0.030 (0.020, 0.040)	0.946
EO#, 10^9^/L	0.11 (0.07, 0.17)	0.12 (0.07, 0.18)	0.228
HGB, g/L	138 (127, 148)	132 (120, 144)	<0.001
LYMPH#, 10^9^/L	1.90 (1.50, 2.30)	1.80 (1.40, 2.20)	<0.001
MONO#, 10^9/L	0.38 (0.31, 0.47)	0.42 (0.33, 0.53)	<0.001
NEUT#, 10^9/L	3.38 (2.70, 4.12)	3.84 (3.00, 4.91)	<0.001
PLT, 10^9^/L	208 (171, 250)	197 (168, 249)	0.259
NLR	1.78 (1.42, 2.28)	2.10 (1.61, 2.99)	<0.001
PLR	110 (87, 140)	119 (91, 151)	0.022
SII, 10^9^/L	364 (267, 495)	435 (302, 637)	<0.001
HBA1C, %	8.60 (7.20, 9.90)	9.60 (8.55, 11.50)	<0.001
GGT, U/L	23 (17, 35)	24 (18, 38)	0.042
TyG	9.18 (8.72, 9.69)	9.51 (9.07, 10.07)	<0.001
AIP	0.12 (-0.09, 0.31)	0.24 (0.00, 0.47)	<0.001
HTN, n (%)	340 (42.2%)	166 (63.8%)	<0.001
Hyperlipidemia, n (%)	465 (57.7%)	146 (56.2%)	0.663
CVD, n (%)	256 (31.8%)	128 (49.2%)	<0.001
MicroVCs, n (%)	530 (65.8%)	212 (81.5%)	<0.001

Continuous values were presented as median [interquartile range]. Categorical values were presented as number (percentage).

A/G, albumin-to-globulin ratio; AIP, atherogenic index of plasma; ALB, albumin; ALP, alkaline phosphatase; BASO#, basophil count; BUN, blood urea nitrogen; CVD, cardiovascular disease; EO#, eosinophil count; GGT, gamma-glutamyl transferase; GLU, fasting glucose; HbA1c, glycated hemoglobin; HDL, high-density lipoprotein cholesterol; HGB, hemoglobin; HTN, hypertension; LDL-C, low-density lipoprotein cholesterol; LYMPH#, lymphocyte count; MicroVCs, microvascular complications; MONO#, monocyte count; NEUT#, neutrophil count; NLR, neutrophil-to-lymphocyte ratio; PAB, prealbumin; PLR, platelet-to-lymphocyte ratio; PLT, platelet count; SII, systemic immune-inflammation index; TBIL, total bilirubin; TG, triglycerides; TP, total protein; TyG, triglyceride-glucose index; UA, uric acid.

### Model comparison and feature selection

**Figure 2:**
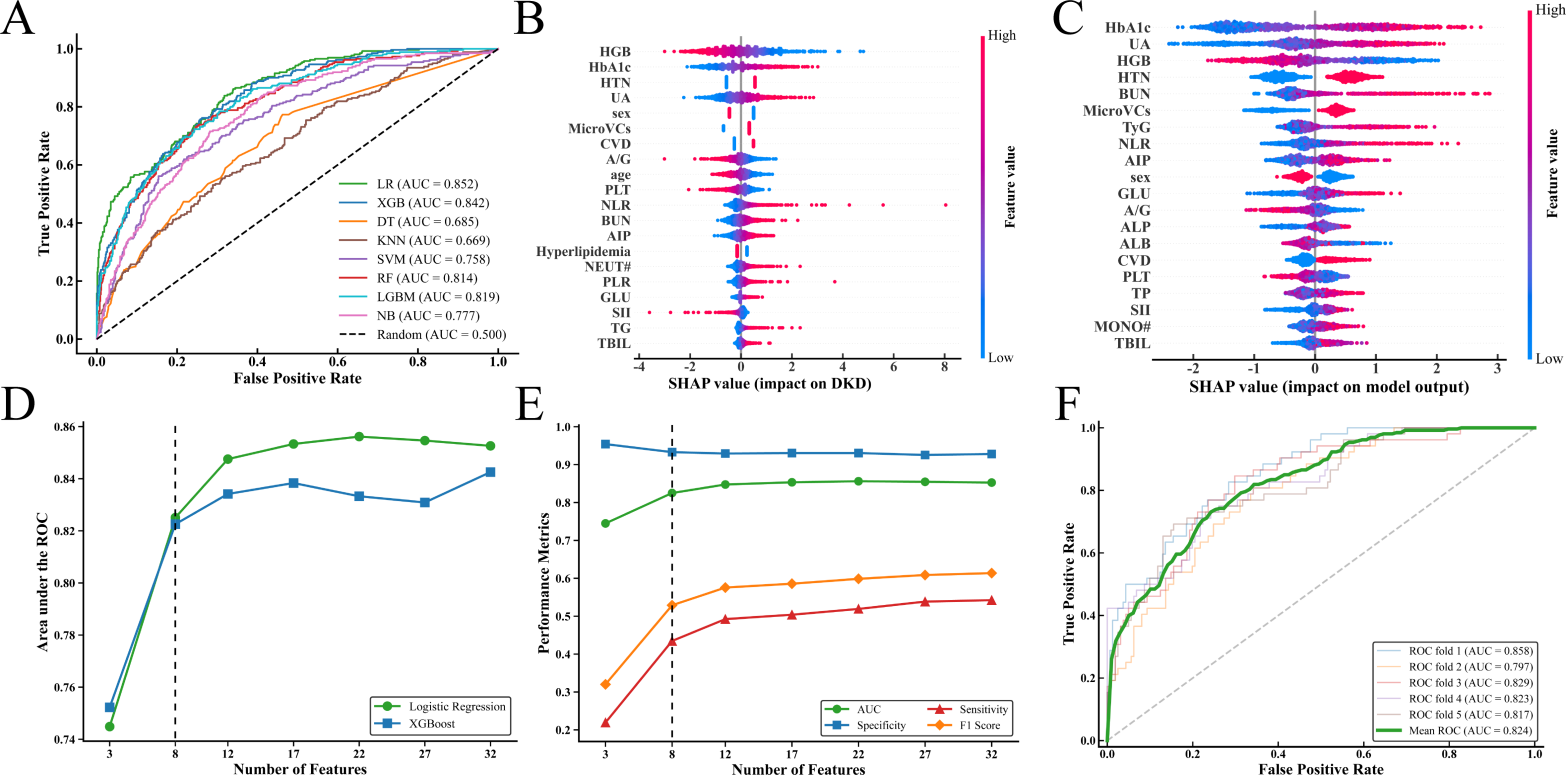
Model comparison and feature selection for occult DKD prediction. **(A)** AUCs of the eight ML models with 32 features. **(B, C)** SHAP feature importance rankings of the LR **(B)** and XGBoost **(C)** models. **(D)** AUCs of the LR and XGBoost models with varied numbers of features. **(E)** AUC, accuracy, sensitivity, specificity, and F1 score of the LR model with varied numbers of features. **(F)** 5-fold cross-validation ROC curve of the final 8-feature LR model.

The initial predictive performance of the eight machine learning algorithms, using the full set of 32 clinlabomics features, is shown in **Figure 2A**. Precision-recall (PR) curves and confusion matrix heatmaps for all eight models are presented in **Supplementary Figure 2** and **S3**. LR and XGBoost demonstrated the highest discriminative ability, with AUC values of 0.852 and 0.842, respectively. Detailed performance metrics for all models, including accuracy and F1 score, are provided in **Supplementary Table 3**. SHAP analysis was then performed to assess global feature importance and identify the key drivers of the two top-performing models. The SHAP-derived importance rankings of the top 20 features for LR and XGBoost are presented in **Figures 2B, C**, respectively. These rankings guided the sequence for subsequent feature reduction.

During the feature reduction process, the LR model retained near-optimal predictive performance, as illustrated in **Figure 2D**. Performance metrics for the LR model across different feature subsets are provided in **Figure 2E** and **Supplementary Table 4**. Sensitivity, specificity, positive predictive value (PPV), negative predictive value (NPV), accuracy, and F1 score were calculated at the optimal cutoff, determined by maximizing the Youden index.

For predicting occult DKD, the full 32-feature LR model significantly outperformed the 3-feature model (ΔAUC = 0.108, *P* < 0.001) and the 7-feature model (ΔAUC = 0.033, *P* = 0.044). However, it showed no significant advantage over the 8-feature model (ΔAUC = 0.027, *P =* 0.094). The 8-feature model also provided substantial net clinical benefit across a wide range of threshold probabilities, comparable to that of the 32-feature model. Additionally, the area under the precision-recall (PR) curve for the 8-feature model was only slightly lower than that of the 32-feature model, indicating that both models offer similarly high clinical utility (**Supplementary Figure 4**). Based on these results, the 8-feature LR model including HGB, HbA1c, HTN, UA, sex, MicroVCs, CVD, and A/G was selected as the final model for all subsequent analyses.

In predicting occult DKD, the final LR model achieved an AUC of 0.824, sensitivity of 0.435, specificity of 0.933, positive predictive value (PPV) of 0.682, negative predictive value (NPV) of 0.837, accuracy of 0.811, and an F1 score of 0.529 (**Figure 2F**).

For the external validation, the final LR model achieved an AUC of 0.786, which was statistically comparable to its cross-validated performance in the training cohort (ΔAUC = 0.038, *P* = 0.103), indicating that the final model maintained robust and consistent predictive power across both cohorts (**Supplementary Figure 5**).

### Model explanation

Because clinicians often hesitate to adopt machine learning models that function as “black boxes,” the SHAP method, together with traditional statistical tools, was used to interpret the output of the final 8-feature LR model. This explanatory framework provided global explanations at the feature level, describing overall model behavior, and local explanations at the individual level, illustrating personalized prediction trajectories.

**Figure 3:**
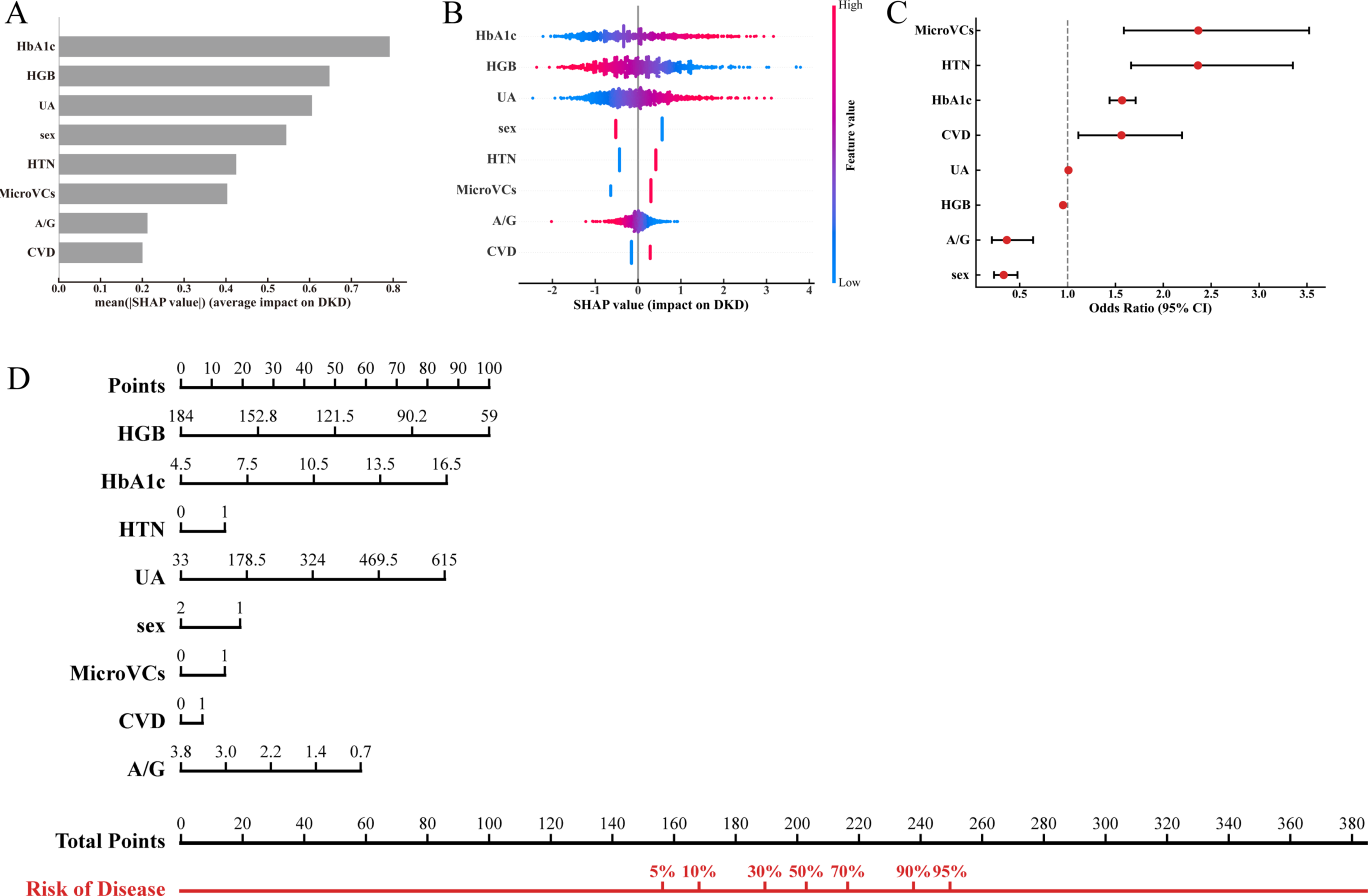
Global explanation of the final 8-feature LR model. **(A, B)** SHAP summary plots displaying the mean absolute SHAP values **(A)** and the distribution of feature impacts on the model output **(B)**. **(C)** Forest plot illustrating the odds ratios (ORs) and 95% confidence intervals (CIs) of the core features. **(D)** Nomogram for predicting the individualized probability of occult DKD.

Globally, SHAP summary plots (**Figures 3A, B**) ranked the contributions of each feature in descending order of importance. HbA1c, HGB, and UA were identified as the top three driving factors for predicting occult DKD. A SHAP beeswarm plot (**Figure 3B**) further illustrated the directional effects of these features on risk. Additionally, a forest plot (**Figure 3C**) integrated AI-derived insights with traditional clinical statistics. The analysis consistently showed that higher HbA1c levels and the presence of MicroVCs, HTN, and CVD were significant independent risk factors (OR > 1), whereas HGB and A/G acted as protective factors (OR < 1), fully consistent with the SHAP findings. Finally, a nomogram (**Figure 3D**) was developed based on the global model weights. By converting the regression formula into an intuitive visual scoring system, the nomogram clearly demonstrates how specific clinical values accumulate “Points” to substantially increase the overall risk of occult DKD.

**Figure 4:**
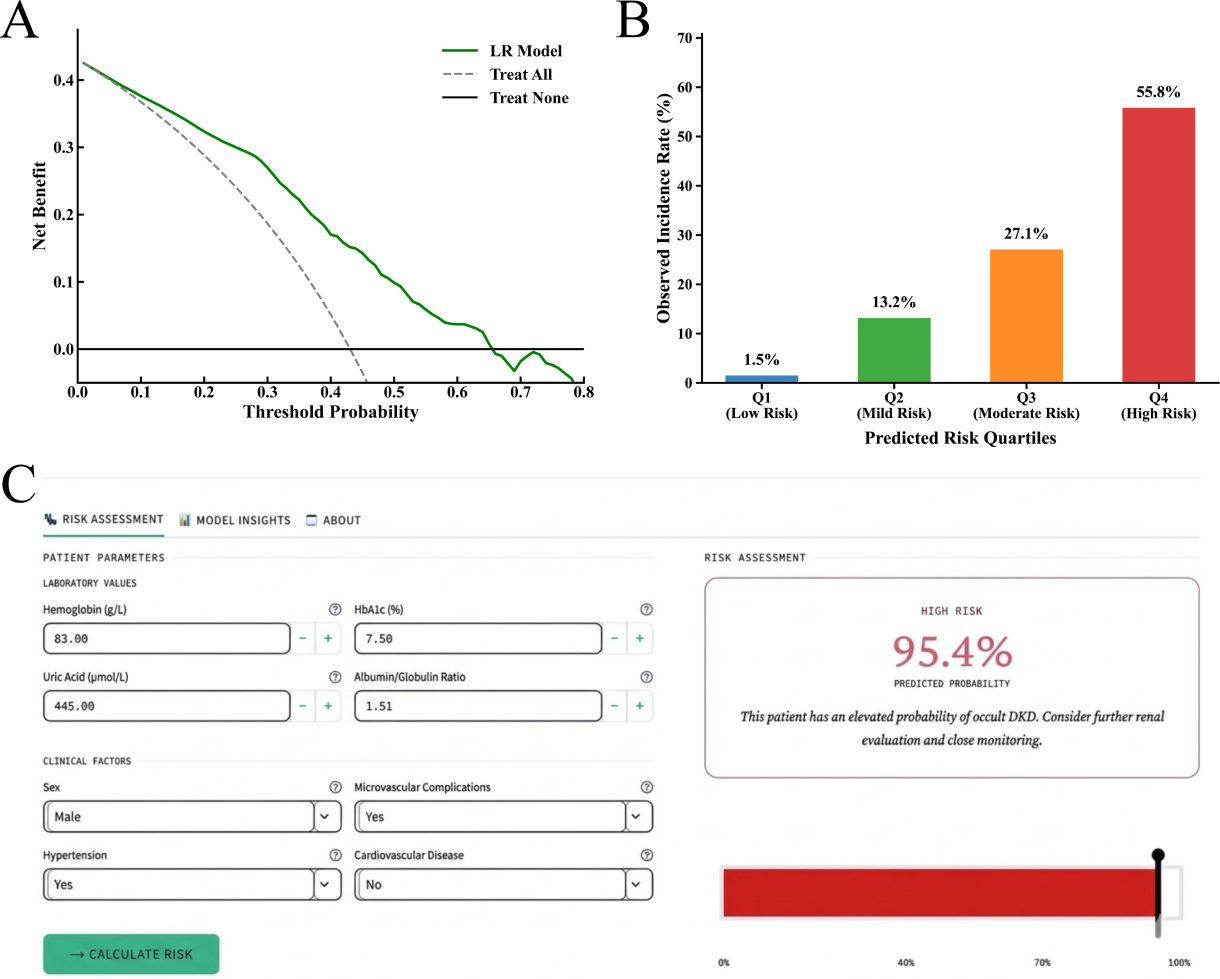
Local explanation of the LR model for individualized prediction. **(A)** SHAP heatmap visualizing the heterogeneous impacts of the 8 features across the entire patient cohort. **(B, C)** SHAP waterfall plots detailing the specific predictive trajectories for a representative high-risk patient positive for occult DKD **(B)** and a low-risk patient negative for occult DKD **(C)**.

In addition, local explanations were used to illustrate how predictions were generated for individual patients based on their specific input data. A SHAP heatmap (**Figure 4A**) visualized the heterogeneous impact of the eight features across the entire cohort. **Figures 4B, C** then presented the predictive trajectories of a representative high-risk patient (positive for occult DKD) and a low-risk patient (negative) using SHAP waterfall plots. In these plots, the actual measured feature values were prominently displayed. In the positive case (**Figure 4B**), elevated values of risk factors such as HbA1c, UA, and the presence of HTN contributed strongly to the prediction of the “occult DKD” class. In contrast, in the negative case (**Figure 4C**), protective features including normal HGB and A/G levels shifted the prediction toward the “non-occult DKD” class. Importantly, even if a patient had a single abnormal indicator, the strong protective effects of other normal features could still result in a low overall risk prediction. This dynamic weighting mechanism clearly demonstrates how individualized risk profiles are formed in clinical practice.

### Clinical application and risk stratification

**Figure 5:**
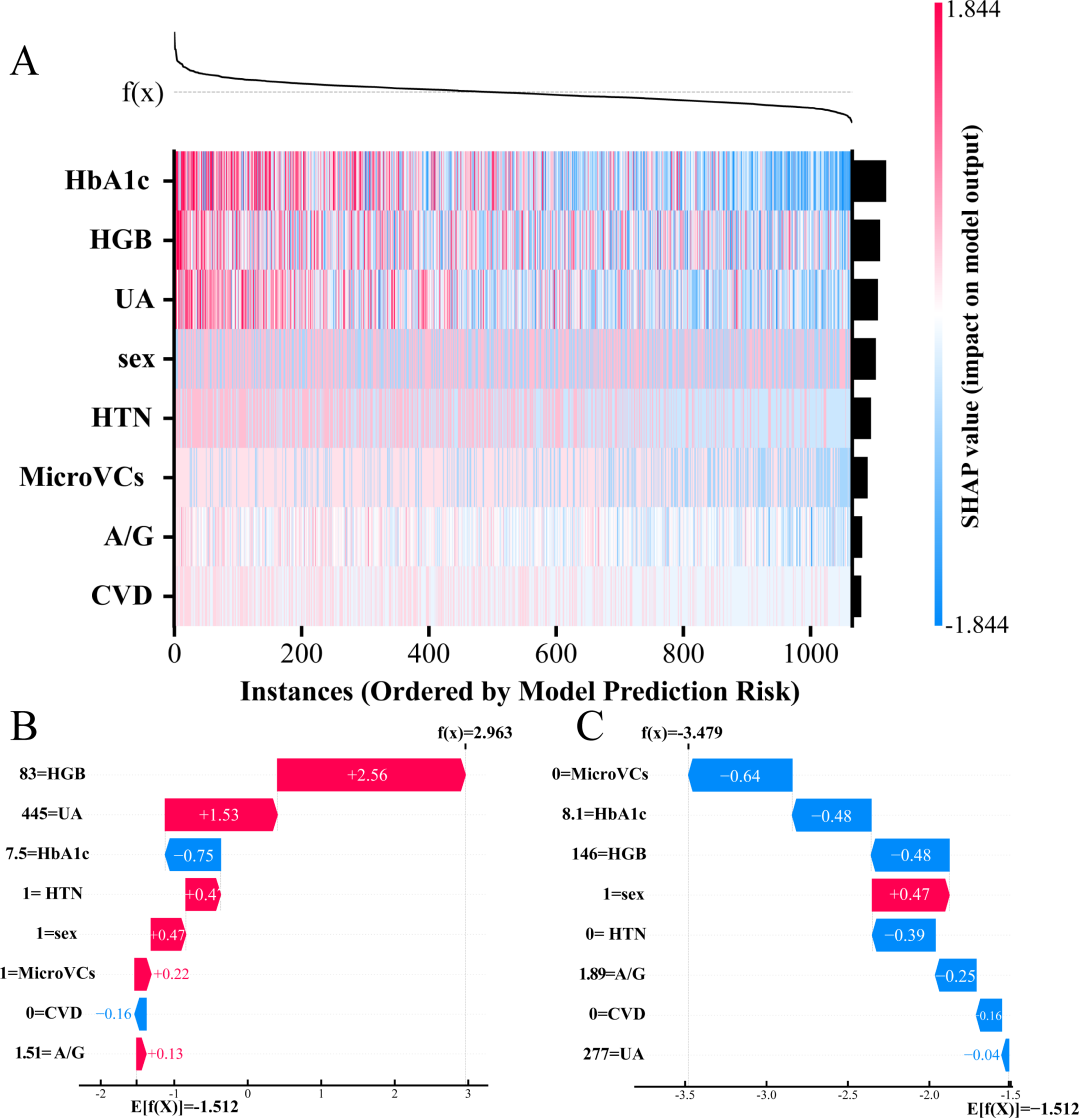
Clinical utility and application of the optimal LR model. **(A)** Net benefits of the LR model by decision curve analysis. **(B)** Observed incidence rates of occult DKD across four predicted risk quartiles. **(C)** User interface of the web-based calculator for individualized risk assessment.

The practical clinical utility of the optimal LR model was evaluated using decision curve analysis (DCA). As shown in **Figure 5A**, the model provided substantial clinical net benefit across a wide range of threshold probabilities compared with the default “treat-all” and “treat-none” strategies, confirming its robustness for clinical decision-making. Risk stratification based on the model’s predicted probabilities revealed a significant, stepwise increase in the observed incidence of occult DKD. Specifically, the actual incidence rates across the four risk quartiles were 1.5% in Q1 (Low Risk), 13.2% in Q2 (Mild Risk), 27.1% in Q3 (Moderate Risk), and 55.8% in Q4 (High Risk) (**Figure 5B**). This clear gradient highlights the model’s strong ability to distinguish high-risk populations from those at lower risk.

Building upon these validated results, the optimal model was deployed as a user-friendly, web-based online calculator to facilitate real-world clinical application. This interactive tool, freely accessible at https://dkd-zhangmengyu.streamlit.app/, allows clinicians to input routine laboratory and clinical parameters and obtain an individualized risk assessment (**Figure 5C**). By providing an intuitive probability gauge and clear risk interpretation, the calculator bridges the gap between complex machine learning algorithms and bedside decision-making, offering a practical solution for early occult DKD screening in primary care settings.

## Discussion

The rising global prevalence of T2DM has made DKD a leading cause of end-stage renal disease worldwide (23). While overt DKD is typically diagnosed using established thresholds for proteinuria and declining eGFR, occult DKD remains a highly insidious phenotype (24, 25). Patients with occult DKD often experience progressive renal structural damage including glomerular basement membrane thickening, podocyte loss, and tubulointerstitial fibrosis long before traditional screening markers such as the UACR show abnormalities (26). Reliance on these late-stage functional indicators inevitably misses the critical window for early prophylactic intervention. Therefore, timely detection and risk stratification are essential to reduce the substantial clinical and socioeconomic burden of diabetic nephropathy. In this multicenter retrospective study, we developed and validated a clinlabomics-based machine learning model specifically designed for the early detection of occult DKD. Using eight readily available routine clinical features, the LR model demonstrated excellent discriminative performance, achieving an AUC of 0.824 in the training cohort and 0.786 in the external validation cohort. It is important to note that predictive metrics such as the positive predictive value (PPV) and the baseline of the precision-recall (PR) curve are inherently dependent on disease prevalence. Consequently, the model’s real-world predictive performance may vary when applied to outpatient or community populations with different baseline risks.

A persistent challenge in current DKD screening, particularly in primary care, is the underuse or delayed implementation of comprehensive biomarker profiling (27). Epidemiological data indicate that routine UACR testing is often inconsistently performed at the community healthcare level. To address this practical limitation, our study leveraged “clinlabomics” routine blood indices and basic medical history that are universally collected during standard diabetic check-ups. Through rigorous dimensionality reduction guided by SHAP importance rankings, we reduced an initial set of 32 variables to an optimized 8-feature subset (HbA1c, UA, HGB, A/G, HTN, sex, MicroVCs, and CVD). This feature reduction maintained an AUC comparable to the full 32-feature model while substantially reducing computational complexity and the economic burden of clinical data collection.

Our interpretability analysis identified HbA1c and UA as the two strongest driving factors for the onset of occult DKD. The pivotal role of HbA1c is biologically well established. Chronic hyperglycemia triggers a cascade of metabolic disturbances, resulting in the accumulation of advanced glycation end-products (AGEs), oxidative stress, and subsequent mesangial matrix expansion (28, 29). Beyond absolute glucose levels, recent large-scale observational studies highlight that long-term visit-to-visit variability of HbA1c independently predicts CKD progression and acute kidney injury, even in patients with well-controlled baseline glucose (30). Therefore, tight and individualized glycemic management remains the cornerstone for delaying glomerular filtration rate decline in early-stage diabetic complications.

Similarly, the identification of UA as a major risk factor aligns with emerging evidence on early tubulointerstitial injury (31). Once considered merely a byproduct of purine metabolism, UA is now recognized as a potent mediator of renal damage. Hyperuricemia induces endothelial dysfunction, thickens preglomerular arterioles, and activates the intrarenal renin-angiotensin system (RAS), promoting glomerular hypertension long before macroscopic damage occurs (32, 33). In the diabetic state, endogenous fructose metabolism further increases tubular UA production, directly contributing to tubulointerstitial injury. Clinical studies have shown that elevated serum UA levels strongly correlate with higher urinary concentrations of kidney injury molecule-1 (KIM-1) and pro-inflammatory cytokines such as TNF-alpha in patients with T2DM, indicating significant tubular damage even in the absence of clinical albuminuria (34).

Conversely, our machine learning model assigned strong protective weights to HGB and the albumin-to-globulin (A/G) ratio. The inverse relationship between HGB levels and occult DKD risk highlights the early interplay between anemia and renal hypoxia. Anemia is increasingly recognized as an early comorbidity in diabetes, often driven by impaired renal oxygen sensing and reduced erythropoietin production, which are closely linked to initial tubulointerstitial fibrosis (35). Lower HGB levels, combined with hypoalbuminemia, have been independently validated as robust predictors of rapid eGFR decline in normoalbuminuric diabetic cohorts (36). The A/G ratio serves as a composite marker reflecting both nutritional status and systemic inflammation. Albumin functions as a negative acute-phase reactant and scavenger of reactive oxygen species (ROS) (37), whereas elevated serum globulins indicate chronic, low-grade immune activation, common in metabolic diseases (38, 39). A declining A/G ratio signals a shift toward a pro-inflammatory state, increasing the susceptibility of the renal microvasculature to diabetic insults.

The inclusion of HTN, CVD, and specific microvascular complications (MicroVCs) in the final model underscores the systemic nature of diabetic end-organ damage. The pathophysiology of diabetic nephropathy is closely linked to systemic vascular disease. Persistent systemic hypertension elevates intra-renal perfusion, causing shear stress and arteriosclerosis in the renal arteries, which accelerates nephron loss (40). The clustering of these macro- and microvascular risk factors within the optimized 8-feature subset supports the view that occult DKD represents a localized renal manifestation of widespread endothelial dysfunction and systemic angiopathy.

A major barrier to the widespread clinical adoption of artificial intelligence in medicine is the inherent “black-box” nature of complex algorithms. Physicians are understandably hesitant to make critical decisions based on opaque mathematical computations. This study addressed this limitation by incorporating both global and local SHAP explanations. The global SHAP summary provided a clear hierarchy of feature importance, while the local SHAP waterfall plots illustrated the dynamic weighting mechanism underlying an individual patient’s risk profile. By translating these AI-driven insights into a traditional forest plot and a visual nomogram, the algorithmic outputs were anchored within well-established epidemiological and statistical principles.

The 8-feature LR model was further translated into a four-tier risk stratification system and an interactive web-based predictor to maximize clinical utility. The risk stratification revealed a sharp, well-calibrated gradient, with observed occult DKD incidence rising from 1.5% in the lowest quartile to 55.8% in the highest quartile. This system enables primary care providers to efficiently allocate healthcare resources and identify patients who require intensive metabolic management or early nephrology referral, even when UACR values remain deceptively normal. The web-based application eliminates complex computations, allowing clinicians to input routine laboratory values and instantly obtain individualized risk assessments. This seamless integration of advanced algorithms into an accessible digital tool represents a significant advance in democratizing precision medicine at the primary care level.

Beyond early screening, our findings carry significant implications for both future clinical practice and fundamental research. Clinically, this predictive model could serve as a dynamic monitoring tool to evaluate therapeutic efficacy. By longitudinally tracking a patient’s risk probability, physicians can quantitatively assess the impact of lifestyle interventions, established renoprotective pharmacotherapies, and emerging multi-target treatments, including Traditional Chinese Medicine (41) and the integrated concept of food–medicine homology (42). Moreover, this dynamic risk assessment aligns with the increasing demand for continuous evaluation of comprehensive cardio–renal risk profiles in patients with diabetes (43). From a fundamental research perspective, the core predictive features identified by our SHAP analysis particularly the A/G ratio, uric acid, and hemoglobin highlight the complex interplay among chronic low-grade inflammation, oxidative stress, and metabolic dysfunction, such as visceral adiposity (44). These clinlabomic indicators suggest that early DKD is driven by systemic biological crosstalk, including metabolic interactions mediated by gut microbiota (45). Collectively, these clinical insights provide potential therapeutic targets for mechanistic studies investigating the initial pathways of diabetic renal injury, encompassing epigenetic regulation (41), alterations in metabolic markers (46, 47), and specific anti-inflammatory signaling pathways, such as modulation of AHR or NF-κB/Nrf2 signaling (48, 49).

Several limitations of this study should be acknowledged. First, the retrospective design inherently introduces selection bias. Importantly, because data were derived from routine electronic medical records, several unmeasured confounders including specific renoprotective medications (e.g., SGLT2 inhibitors, GLP-1 receptor agonists, and RAAS inhibitors) and detailed clinical parameters (e.g., BMI, diabetes duration, smoking history, and longitudinal blood pressure measurements) were unavailable, precluding further sensitivity analyses. Second, the cross-sectional nature of the laboratory data provides only a single time point, limiting the assessment of longitudinal biomarker variability. Third, although the model is intended to support primary care screening, both cohorts consisted of hospitalized patients at tertiary centers. As hospitalized populations generally exhibit higher comorbidity burdens than community populations, the generalizability of our model to routine primary care settings requires further prospective validation. Finally, the study population was exclusively Chinese, and extrapolation to other ethnic groups should be undertaken with caution. Future large-scale, multinational outpatient studies are needed to confirm the clinical utility of this web-based tool.

## Conclusion

The clinlabomics-based machine learning framework developed in this study offers a highly accurate, transparent, and practical tool for the early detection of occult DKD. By relying solely on widely available clinical features, it overcomes the diagnostic delays associated with traditional proteinuria screening. The integration of SHAP interpretability, a validated risk stratification system, and a rapid web-based calculator provides primary care physicians with an actionable decision-support tool. Implementing this targeted approach in routine practice has the potential to shift DKD management from reactive interventions to proactive, personalized care.

## Data Availability

The raw data supporting the conclusions of this article will be made available by the authors, without undue reservation.
