# Supplementary material for: Developing and validating a clinlabomics-based machine-learning model for early detection of occult diabetic kidney disease: implications for primary care screening
Source: Front Endocrinol (Lausanne). 2026 Apr 27;17:1835866. doi: 10.3389/fendo.2026.1835866 (PMC13158108; doi:10.3389/fendo.2026.1835866)
Supplement: Supplementary file 1 [file DataSheet1.docx]

**Table S1. Detailed descriptions, abbreviations, and measurement units of the candidate clinlabomics features used for model development.**

| **Variables** | **Training cohort** | **Percentage of**  **missing data,** | **External validation**  **cohort** | **Percentage of**  **missing data,** |
| --- | --- | --- | --- | --- |
| Male, n (%) | 1066 | 0 | 850 | 0 |
| age, year | 1066 | 0 | 850 | 0 |
| BUN, mmol/L | 1066 | 0 | 850 | 0 |
| UA, μmol/L | 1066 | 0 | 850 | 0 |
| BASO#, 10^9^/L | 1066 | 0 | 850 | 0 |
| BASO, % | 1066 | 0 | 850 | 0 |
| EO#, 10^9^/L | 1066 | 0 | 850 | 0 |
| EO, % | 1066 | 0 | 850 | 0 |
| HCT, % | 1066 | 0 | 850 | 0 |
| HGB, g/L | 1066 | 0 | 850 | 0 |
| LYMPH#, 10^9^/L | 1066 | 0 | 850 | 0 |
| LYMPH, % | 1066 | 0 | 850 | 0 |
| MCH, pg | 1066 | 0 | 850 | 0 |
| MCHC, g/L | 1066 | 0 | 850 | 0 |
| MCV, fL | 1066 | 0 | 850 | 0 |
| MONO#, 10^9/L | 1066 | 0 | 850 | 0 |
| MONO, % | 1066 | 0 | 850 | 0 |
| NEUT#, 10^9/L | 1066 | 0 | 850 | 0 |
| NEUT, % | 1066 | 0 | 850 | 0 |
| PLT, 10^9^/L | 1066 | 0 | 850 | 0 |
| RBC, 10^12^/L | 1066 | 0 | 850 | 0 |
| RDW-CV, % | 1066 | 0 | 850 | 0 |
| RDW-SD, fL | 1066 | 0 | 850 | 0 |
| WBC, 10^9^/L | 1066 | 0 | 850 | 0 |
| MPV, fL | 1062 | 0.3 | 849 | 0.1 |
| P-LCR, % | 1062 | 0.3 | 849 | 0.1 |
| PCT, % | 1062 | 0.3 | 849 | 0.1 |
| PDW, fL | 1062 | 0.3 | 849 | 0.1 |
| A/G | 1053 | 1.2 | 842 | 0.9 |
| ALB, g/L | 1053 | 1.2 | 842 | 0.9 |
| ALP, U/L | 1053 | 1.2 | 842 | 0.9 |
| ALT, U/L | 1053 | 1.2 | 842 | 0.9 |
| AST, U/L | 1053 | 1.2 | 842 | 0.9 |
| DBIL, μmol/L | 1053 | 1.2 | 842 | 0.9 |
| GGT, U/L | 1053 | 1.2 | 842 | 0.9 |
| GLB, g/L | 1053 | 1.2 | 842 | 0.9 |
| IBIL, μmol/L | 1053 | 1.2 | 842 | 0.9 |
| TBIL, μmol/L | 1053 | 1.2 | 842 | 0.9 |
| TP, g/L | 1053 | 1.2 | 842 | 0.9 |
| Cl, mmol/L | 1044 | 2.0 | 823 | 3.1 |
| K, mmol/L | 1044 | 2.0 | 823 | 3.1 |
| Na, mmol/L | 1044 | 2.0 | 823 | 3.1 |
| ECO2, mmol/L | 1041 | 2.3 | 844 | 0.6 |
| ZCA, mmol/L | 1041 | 2.3 | 844 | 0.6 |
| HDL, mmol/L | 1040 | 2.4 | 841 | 1.0 |
| LDL-C, mmol/L | 1040 | 2.4 | 841 | 1.0 |
| TG, mmol/L | 1040 | 2.4 | 838 | 1.4 |
| HBA1C, % | 992 | 6.9 | 838 | 1.4 |
| CHOL, mmol/L | 1039 | 2.5 | 838 | 1.4 |
| GLU, mmol/L | 943 | 11.5 | 802 | 5.6 |
| PAB, mg/L | 887 | 16.7 | 802 | 5.6 |
| TBA, μmol/L | 887 | 16.7 | 802 | 5.6 |
| HTN | 1047 | 1.7 | 842 | 0.9 |
| Hyperlipidemia | 1047 | 1.7 | 842 | 0.9 |
| CVD | 1047 | 1.7 | 842 | 0.9 |
| MicroVCs | 1047 | 1.7 | 842 | 0.9 |
| NLR | 1066 | 0 | 850 | 0 |
| PLR | 1066 | 0 | 850 | 0 |
| SII, 10^9^/L | 1066 | 0 | 850 | 0 |
| LDH, U/L | 661 | 37.9 | 787 | 7.4 |
| ASTm, U/L | 652 | 38.8 | 787 | 7.4 |
| TyG | 1066 | 0 | 850 | 0 |
| AIP | 1066 | 0 | 850 | 0 |
| eGFR^a^, mL/min/1.73 m^2^ | 1066 | 0 | 850 | 0 |

^a^The eGFR was calculated based on the CKD-EPI (Chronic Kidney Disease Epidemiology Collaboration) equation.

A/G, albumin-to-globulin ratio; AIP, atherogenic index of plasma [calculated as log10(TG/HDL-C)]; ALB, albumin; ALP, alkaline phosphatase; ALT, alanine aminotransferase; AST, aspartate aminotransferase; ASTm, mitochondrial aspartate aminotransferase; BASO, basophil percentage; BASO#, basophil count; BUN, blood urea nitrogen; CHOL, total cholesterol; Cl, chloride; CVD, cardiovascular disease; DBIL, direct bilirubin; ECO2, total carbon dioxide; eGFR, estimated glomerular filtration rate; EO, eosinophil percentage; EO#, eosinophil count; GGT, gamma-glutamyl transferase; GLB, globulin; GLU, fasting glucose; HbA1c, glycated hemoglobin; HCT, hematocrit; HDL-C, high-density lipoprotein cholesterol; HGB, hemoglobin; HTN, hypertension; IBIL, indirect bilirubin; K, potassium; LDH, lactate dehydrogenase; LDL-C, low-density lipoprotein cholesterol; LYMPH, lymphocyte percentage; LYMPH#, lymphocyte count; MCH, mean corpuscular hemoglobin; MCHC, mean corpuscular hemoglobin concentration; MCV, mean corpuscular volume; MicroVCs, microvascular complications; MONO, monocyte percentage; MONO#, monocyte count; MPV, mean platelet volume; Na, sodium; NEUT, neutrophil percentage; NEUT#, neutrophil count; NLR, neutrophil-to-lymphocyte ratio [calculated as NEUT#/LYMPH#]; P-LCR, platelet large cell ratio; PAB, prealbumin; PCT, plateletcrit; PDW, platelet distribution width; PLR, platelet-to-lymphocyte ratio [calculated as PLT/LYMPH#]; PLT, platelet count; RBC, red blood cell count; RDW-CV, red blood cell distribution width-coefficient of variation; RDW-SD, red blood cell distribution width-standard deviation; SII, systemic immune-inflammation index [calculated as (PLT×NEUT#)/LYMPH#]; TBA, total bile acids; TBIL, total bilirubin; TG, triglycerides; TP, total protein; TyG, triglyceride-glucose index [calculated as ln(TG×GLU/2)]; UA, uric acid; WBC, white blood cell count; ZCA, total calcium.

**Table S2.** **Detailed descriptions, abbreviations, and measurement units of the 32 candidate clinlabomics features in external validation cohort.**

| **Characteristic** | **Label** | | **p-value** |
| --- | --- | --- | --- |
|  | **Non-occult DKD  N = 484** | **occult DKD  N = 366** |  |
| age, year | 61 (53, 70) | 60 (51, 69) | 0.3802 |
| Male, n (%) | 283 (58.5%) | 261 (71.3%) | <0.001 |
| A/G, | 1.90 (1.80, 2.01) | 1.87 (1.68, 1.90) | <0.001 |
| ALB, g/L | 43.21 (42.24, 44.25) | 43.19 (41.90, 43.28) | 0.017 |
| ALP, U/L | 75 (69, 81) | 76 (72, 83) | 0.007 |
| BUN, mmol/L | 5.99 (5.40, 6.60) | 6.37 (5.82, 7.26) | <0.001 |
| GLU, mmol/L | 8.39 (6.82, 9.08) | 8.49 (7.51, 10.02) | <0.001 |
| HDL, mmol/L | 1.08 (0.96, 1.16) | 1.06 (0.96, 1.13) | 0.007 |
| LDL-C, mmol/L | 2.44 (2.21, 2.89) | 2.43 (2.28, 2.85) | 0.568 |
| PAB, mg/L | 245 (228, 266) | 258 (235, 268) | <0.001 |
| TBIL, μmol/L | 13.2 (11.6, 15.0) | 13.0 (11.3, 14.2) | 0.010 |
| TG, mmol/L | 1.41 (1.18, 1.59) | 1.43 (1.32, 1.73) | 0.001 |
| TP, g/L | 66.0 (64.5, 68.0) | 66.0 (65.7, 67.3) | 0.148 |
| UA, μmol/L | 293 (257, 344) | 144 (128, 151) | <0.001 |
| BASO#, 10^9^/L | 0.030 (0.025, 0.039) | 0.030 (0.027, 0.036) | 0.862 |
| EO#, 10^9^/L | 0.14 (0.11, 0.17) | 0.15 (0.13, 0.17) | 0.026 |
| HGB, g/L | 140 (128, 149) | 338 (282, 362) | <0.001 |
| LYMPH#, 10^9^/L | 1.80 (1.67, 2.00) | 1.74 (1.67, 1.93) | 0.059 |
| MONO#, 10^9/L | 0.40 (0.38, 0.46) | 0.41 (0.39, 0.47) | 0.035 |
| NEUT#, 10^9/L | 3.69 (3.16, 4.08) | 3.88 (3.39, 4.13) | <0.001 |
| PLT, 10^9^/L | 201 (183, 216) | 211 (194, 217) | 0.002 |
| NLR, | 2.05 (1.69, 2.23) | 2.08 (1.99, 2.28) | <0.001 |
| PLR, | 111 (100, 126) | 115 (106, 127) | 0.003 |
| SII, 10^9^/L | 424 (319, 440) | 429 (417, 454) | <0.001 |
| HBA1C, % | 7.98 (7.10, 8.74) | 8.25 (7.57, 9.50) | <0.001 |
| GGT, U/L | 20 (15, 25) | 21 (17, 28) | <0.001 |
| TyG, | 9.18 (8.86, 9.48) | 9.19 (8.96, 9.73) | <0.001 |
| AIP, | 0.12 (0.02, 0.19) | 0.13 (0.10, 0.25) | <0.001 |
| HTN, n (%) | 96 (19.8%) | 95 (26.0%) | 0.034 |
| Hyperlipidemia, n (%) | 87（17.9%） | 59（16.1%） | 0.468 |
| CVD, n (%) | 90 (18.6%) | 42 (11.5%) | 0.005 |
| MicroVCs, n (%) | 50 (10.3%) | 160 (43.7%) | <0.001 |

Continuous values were presented as median [interquartile range]. Categorical values were presented as number (percentage).

A/G, albumin-to-globulin ratio; AIP, atherogenic index of plasma; ALB, albumin; ALP, alkaline phosphatase; BASO#, basophil count; BUN, blood urea nitrogen; CVD, cardiovascular disease; EO#, eosinophil count; GGT, gamma-glutamyl transferase; GLU, fasting glucose; HbA1c, glycated hemoglobin; HDL, high-density lipoprotein cholesterol; HGB, hemoglobin; HTN, hypertension; LDL-C, low-density lipoprotein cholesterol; LYMPH#, lymphocyte count; MicroVCs, microvascular complications; MONO#, monocyte count; NEUT#, neutrophil count; NLR, neutrophil-to-lymphocyte ratio; PAB, prealbumin; PLR, platelet-to-lymphocyte ratio; PLT, platelet count; SII, systemic immune-inflammation index; TBIL, total bilirubin; TG, triglycerides; TP, total protein; TyG, triglyceride-glucose index; UA, uric acid.

**Table S3. Performance of the ML models for occult DKD prediction.**

| **Model** | **AUC** | **Accuracy** | **Sensitivity** | **Specificity** | **PPV** | **NPV** | **F1 Score** |
| --- | --- | --- | --- | --- | --- | --- | --- |
| LR | 0.852 | 0.828 | 0.523 | 0.927 | 0.701 | 0.858 | 0.597 |
| XGBoost | 0.842 | 0.807 | 0.412 | 0.937 | 0.678 | 0.832 | 0.510 |
| LGBM | 0.821 | 0.808 | 0.431 | 0.929 | 0.668 | 0.835 | 0.522 |
| RF | 0.814 | 0.801 | 0.250 | 0.979 | 0.803 | 0.802 | 0.377 |
| SVM | 0.760 | 0.770 | 0.169 | 0.964 | 0.591 | 0.783 | 0.260 |
| NB | 0.776 | 0.771 | 0.377 | 0.898 | 0.547 | 0.817 | 0.446 |
| DT | 0.685 | 0.745 | 0.300 | 0.888 | 0.477 | 0.798 | 0.360 |
| KNN | 0.668 | 0.772 | 0.088 | 0.993 | 0.801 | 0.772 | 0.157 |

DKD diabetic kidney disease; AUC, area under the receiver operating characteristic curve; DT, decision tree; KNN, k-nearest neighbor; LGBM, light gradient boosting machine; LR, logistic regression; NB, naive Bayes; NPV, negative predictive value; PPV, positive predictive value; RF, random forest; SVM, support vector machine; XGBoost, extreme gradient boosting.

**Table S4. Performance of the LR model with varied numbers of features for DKD.**

| **Feature numbers** | **AUC** | **Accuracy** | **Sensitivity** | **Specificity** | **F1_Score** | **PPV** | **NPV** |
| --- | --- | --- | --- | --- | --- | --- | --- |
| 32 | 0.852 | 0.834 | 0.542 | 0.928 | 0.614 | 0.712 | 0.863 |
| 31 | 0.852 | 0.829 | 0.535 | 0.924 | 0.604 | 0.700 | 0.860 |
| 30 | 0.852 | 0.828 | 0.535 | 0.923 | 0.603 | 0.695 | 0.860 |
| 29 | 0.852 | 0.831 | 0.538 | 0.926 | 0.609 | 0.702 | 0.861 |
| 28 | 0.853 | 0.831 | 0.535 | 0.927 | 0.607 | 0.704 | 0.861 |
| 27 | 0.855 | 0.831 | 0.538 | 0.926 | 0.609 | 0.704 | 0.862 |
| 26 | 0.855 | 0.827 | 0.527 | 0.924 | 0.598 | 0.697 | 0.858 |
| 25 | 0.856 | 0.829 | 0.527 | 0.927 | 0.600 | 0.704 | 0.859 |
| 24 | 0.856 | 0.830 | 0.531 | 0.927 | 0.604 | 0.705 | 0.860 |
| 23 | 0.856 | 0.829 | 0.519 | 0.929 | 0.597 | 0.706 | 0.857 |
| 22 | 0.856 | 0.830 | 0.519 | 0.931 | 0.599 | 0.709 | 0.857 |
| 21 | 0.857 | 0.829 | 0.519 | 0.929 | 0.597 | 0.706 | 0.857 |
| 20 | 0.857 | 0.830 | 0.519 | 0.930 | 0.599 | 0.713 | 0.857 |
| 19 | 0.858 | 0.830 | 0.527 | 0.928 | 0.601 | 0.708 | 0.859 |
| 18 | 0.855 | 0.831 | 0.523 | 0.931 | 0.601 | 0.711 | 0.858 |
| 17 | 0.853 | 0.826 | 0.504 | 0.931 | 0.586 | 0.703 | 0.853 |
| 16 | 0.854 | 0.828 | 0.500 | 0.934 | 0.587 | 0.715 | 0.853 |
| 15 | 0.854 | 0.827 | 0.500 | 0.933 | 0.585 | 0.711 | 0.853 |
| 14 | 0.855 | 0.827 | 0.508 | 0.930 | 0.589 | 0.706 | 0.854 |
| 13 | 0.856 | 0.825 | 0.515 | 0.924 | 0.589 | 0.687 | 0.855 |
| 12 | 0.848 | 0.823 | 0.492 | 0.929 | 0.576 | 0.694 | 0.850 |
| 11 | 0.842 | 0.826 | 0.492 | 0.933 | 0.580 | 0.707 | 0.851 |
| 10 | 0.845 | 0.821 | 0.477 | 0.932 | 0.566 | 0.700 | 0.847 |
| 9 | 0.843 | 0.820 | 0.477 | 0.930 | 0.565 | 0.699 | 0.846 |
| 8 | 0.825 | 0.811 | 0.435 | 0.933 | 0.529 | 0.680 | 0.837 |
| 7 | 0.819 | 0.812 | 0.431 | 0.935 | 0.528 | 0.686 | 0.836 |
| 6 | 0.819 | 0.808 | 0.415 | 0.934 | 0.513 | 0.675 | 0.832 |
| 5 | 0.810 | 0.805 | 0.373 | 0.944 | 0.481 | 0.681 | 0.824 |
| 4 | 0.795 | 0.796 | 0.354 | 0.938 | 0.456 | 0.647 | 0.818 |
| 3 | 0.745 | 0.775 | 0.219 | 0.954 | 0.320 | 0.606 | 0.791 |
| 2 | 0.707 | 0.776 | 0.173 | 0.970 | 0.272 | 0.650 | 0.784 |
| 1 | 0.604 | 0.764 | 0.035 | 0.999 | 0.065 | 0.600 | 0.762 |

The indexes represented the performance of the LR model with varied numbers of features in the training cohort.

DKD diabetic kidney disease; AUC, area under the receiver operating characteristic curve; NPV: negative predictive value; PPV: positive predictive value; RF: random forest.

**Table S5. Machine Learning Model Configuration Details.**

| **Model** | **Library & Version** | **Preprocessing Pipeline** | **Hyperparameter Search Space**  **(Grid Search)** | **Class Imbalance Handling** | **Random Seed** |
| --- | --- | --- | --- | --- | --- |
| DT (Decision Tree) | scikit-learn v1.3.0 | Label encoding for categorical variables; no feature scaling required | criterion: ['gini', 'entropy']; max_depth: [3, 5, 7, 10, None]; min_samples_split: [2, 5, 10]; min_samples_leaf: [1, 2, 4]; max_features: ['sqrt', 'log2', None] | class_weight = 'balanced' | 42 |
| KNN (K-Nearest Neighbor) | scikit-learn v1.3.0 | StandardScaler (zero mean, unit variance); label encoding for categorical variables | n_neighbors: [3, 5, 7, 9, 11, 15]; weights: ['uniform', 'distance']; metric: ['euclidean', 'manhattan', 'minkowski']; p: [1, 2] | SMOTE applied within training folds only (imbalanced-learn v0.11.0; k_neighbors=5) | 42 |
| LightGBM | lightgbm v4.1.0 | Label encoding; missing values handled natively by LightGBM | num_leaves: [31, 63, 127]; max_depth: [5, 7, 10, -1]; learning_rate: [0.01, 0.05, 0.1]; n_estimators: [100, 300, 500]; min_child_samples: [10, 20, 50]; subsample: [0.7, 0.8, 1.0]; colsample_bytree: [0.7, 0.8, 1.0]; reg_alpha: [0, 0.1]; reg_lambda: [0, 0.1] | scale_pos_weight = n_negative / n_positive | 42 |
| LR (Logistic Regression) | scikit-learn v1.3.0 | StandardScaler; label encoding; one-hot encoding for nominal variables | C: [0.001, 0.01, 0.1, 1, 10, 100]; penalty: ['l1', 'l2', 'elasticnet']; solver: ['liblinear', 'saga']; max_iter: 1000; l1_ratio: [0.2, 0.5, 0.8] (elasticnet only) | class_weight = 'balanced' | 42 |
| NB (Naive Bayes) | scikit-learn v1.3.0 | Label encoding; MinMaxScaler to ensure non-negative values; no feature selection applied | GaussianNB -- var_smoothing: [1e-11, 1e-10, 1e-9, 1e-8, 1e-7]; BernoulliNB -- alpha: [0.01, 0.1, 0.5, 1.0]; binarize: [0.0, 0.3, 0.5] | Manual oversampling (random duplication of minority class) within training folds only | 42 |
| RF (Random Forest) | scikit-learn v1.3.0 | Label encoding; no feature scaling required | n_estimators: [100, 200, 500]; max_depth: [5, 10, 20, None]; min_samples_split: [2, 5, 10]; min_samples_leaf: [1, 2, 4]; max_features: ['sqrt', 'log2']; bootstrap: [True, False] | class_weight = 'balanced_subsample' | 42 |
| SVM (Support Vector Machine) | scikit-learn v1.3.0 | StandardScaler; label encoding; one-hot encoding for nominal variables | C: [0.1, 1, 10, 100]; kernel: ['linear', 'rbf', 'poly']; gamma: ['scale', 'auto', 0.001, 0.01] (rbf/poly); degree: [2, 3] (poly); coef0: [0, 1] (poly) | class_weight = 'balanced' | 42 |
| XGBoost | xgboost v2.0.0 | Label encoding; missing values handled natively by XGBoost | n_estimators: [100, 300, 500]; max_depth: [3, 5, 7]; learning_rate: [0.01, 0.05, 0.1]; subsample: [0.7, 0.8, 1.0]; colsample_bytree: [0.7, 0.8, 1.0]; min_child_weight: [1, 3, 5]; gamma: [0, 0.1, 0.3]; reg_alpha: [0, 0.1]; reg_lambda: [1, 1.5] | scale_pos_weight = n_negative / n_positive | 42 |

Detailed specifications for all eight classifiers, covering library versions, preprocessing pipelines, hyperparameter search spaces, class imbalance handling strategies, and random seeds. All models were optimized via grid search within each training fold of a 5-fold stratified cross-validation framework.

**Figure S1. Heat map of Spearman’s correlation analyses among variables.**


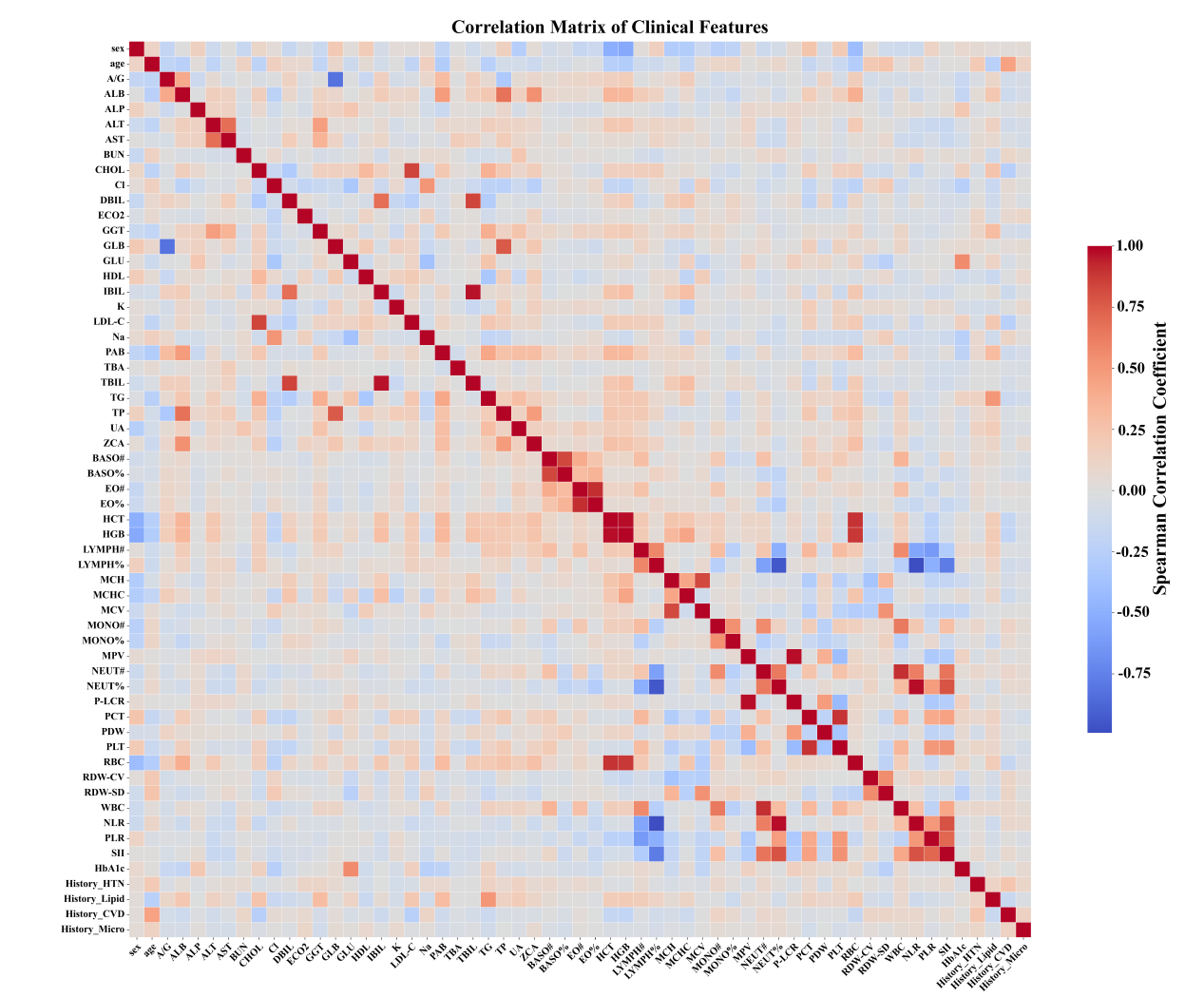


Variables in Supplemental Table S1 (except variables with a missing percentage exceeding 25%) were chosen for the Spearman’s correlation analyses, and the correlation coefficient values were displayed as a heat map.

**Figure S2: PR Curves for predicting occult DKD using 8 machine learning models.**


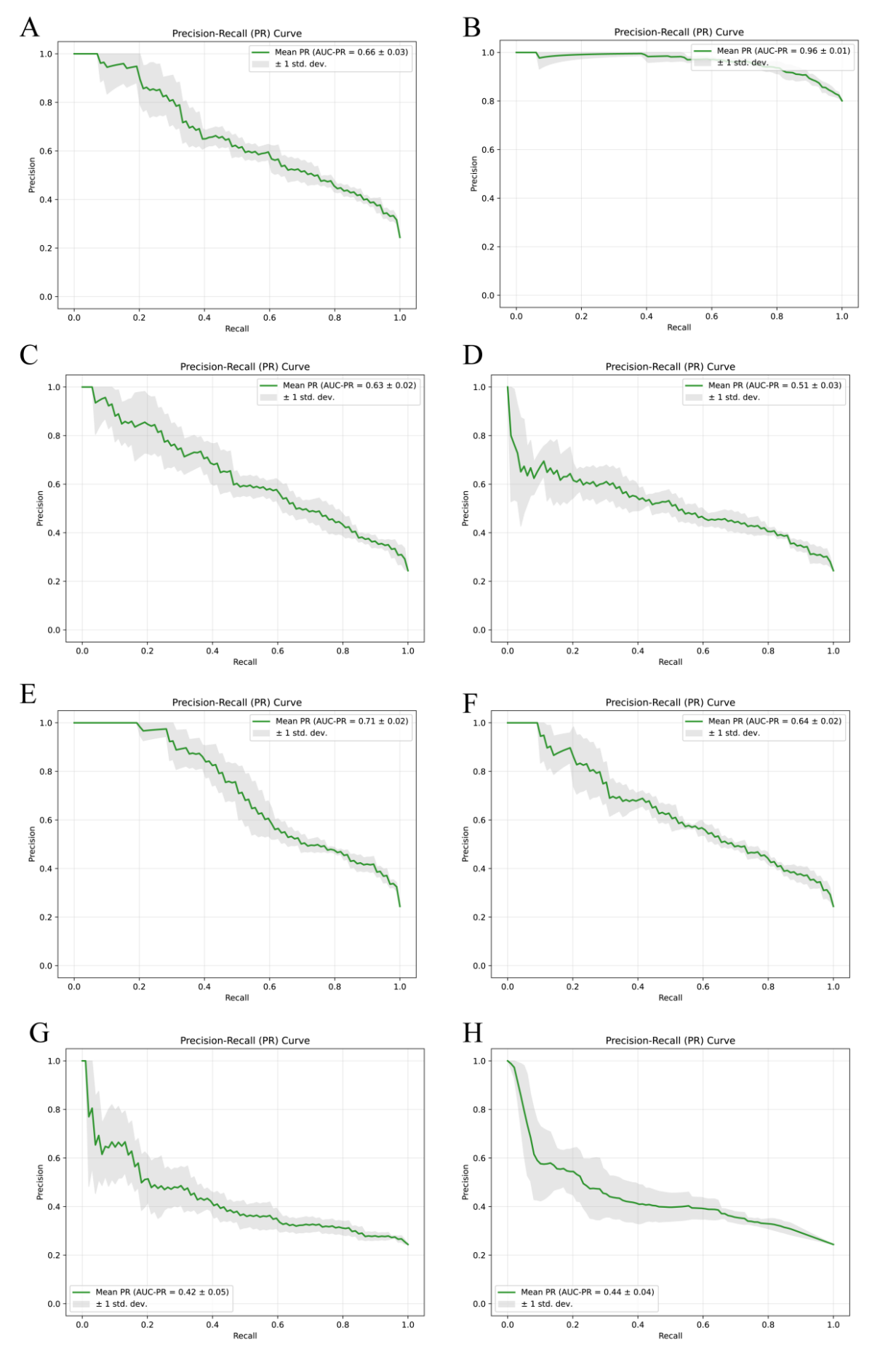


PR curves of the respective models: (A) XGB, (B) SVM, (C) RF, (D) NB, (E) LR, (F) LGBM, (G) KNN, and (H) DT.

**Figure S3: Confusion matrix heat maps for predicting occult DKD using 8 machine learning models.**


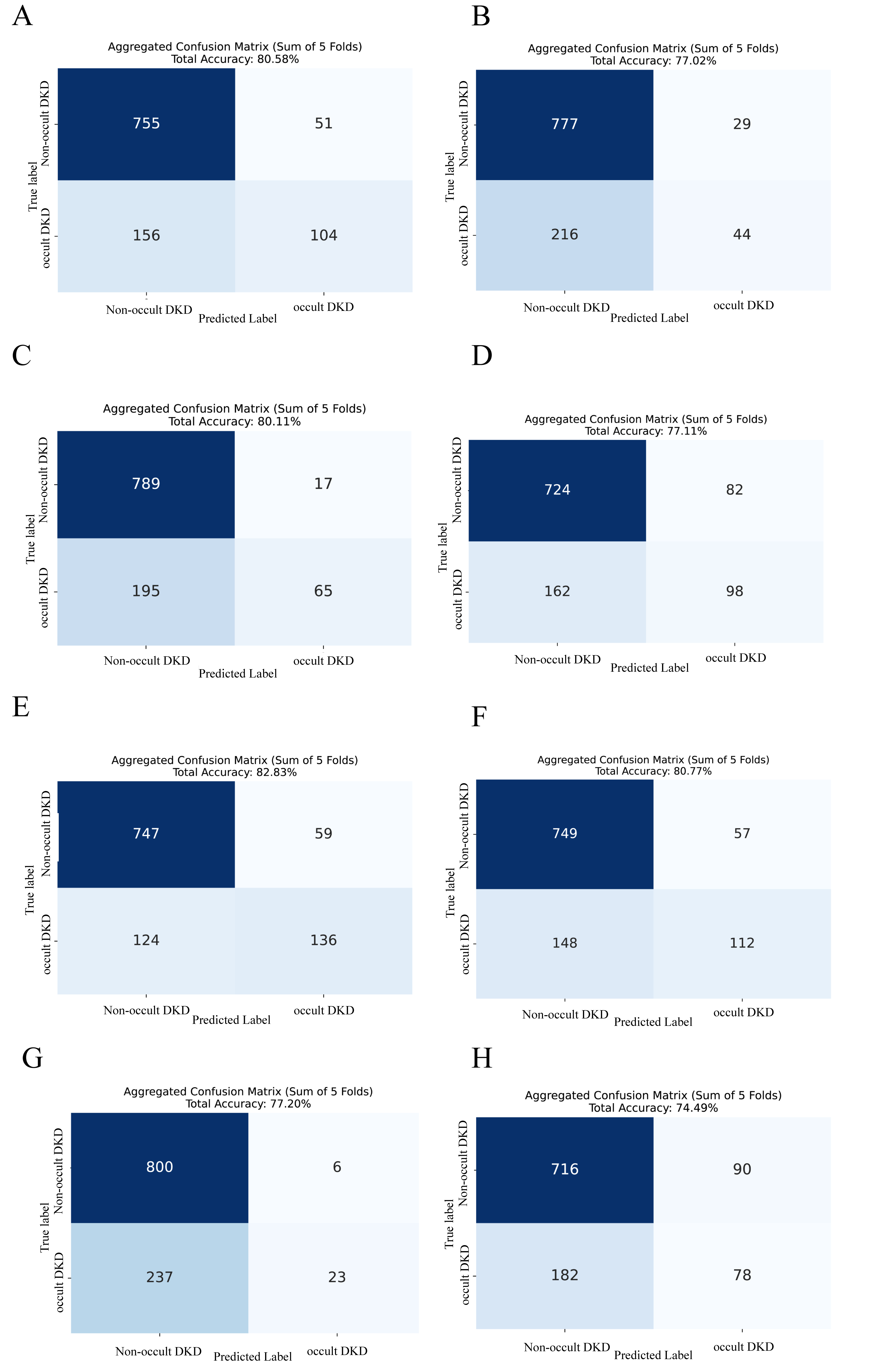


Confusion matrix heat maps of the respective models: (A) XGB, (B) SVM, (C) RF, (D) NB, (E) LR, (F) LGBM, (G) KNN, and (H) DT.

**Figure S4. Predictive performance of the LR model after reducing features**.


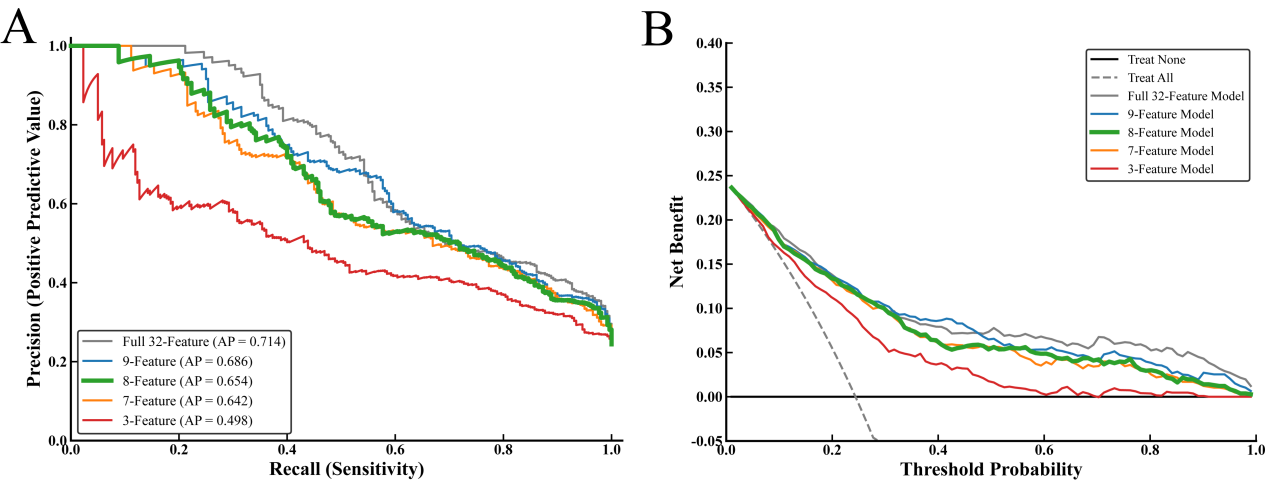


DCA curves(A) and P-R curves(B) of the LR model with different features.These plots represented the predictive performance in the training cohort.

AUC, area under the receiver operating characteristic curve; DCA: decision curve analysis; P-R: precision-recall; LR: logistic regression.

**Figure S5. Predictive performance of the final LR model in the external validation cohort.**

**
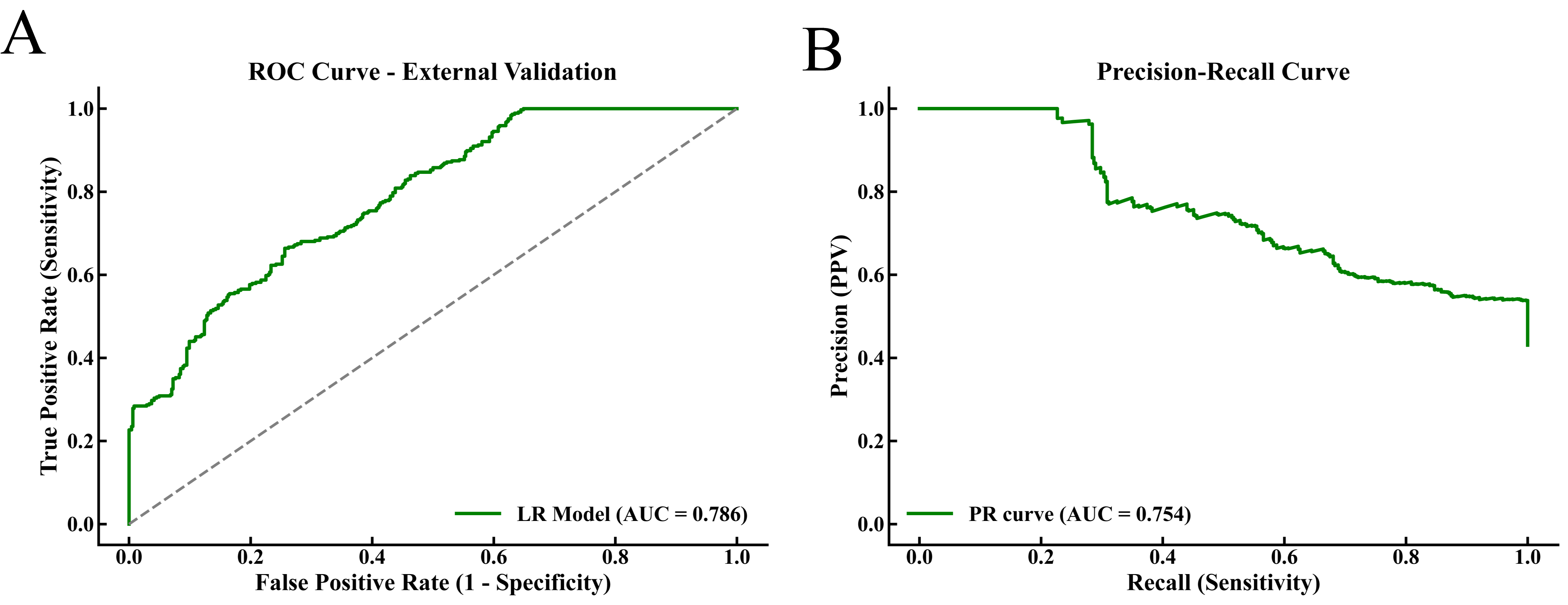
**

(A) The receiver operating characteristic (ROC) curve demonstrating the discrimination ability of the model. (B) The precision-recall (PR) curve illustrating the trade-off between precision and recall for predicting occult diabetic kidney disease (ODKD).
